# Successful ventilator weaning following vascular bypass in patient with vascular tracheobronchial compression

**DOI:** 10.1002/rcr2.320

**Published:** 2018-04-20

**Authors:** Kei Sonehara, Toshitaka Shomura, Masanori Yasuo, Atsuhito Ushiki, Hiroshi Yamamoto, Masayuki Hanaoka

**Affiliations:** ^1^ The First Department of Internal Medicine Shinshu University School of Medicine Matsumoto Japan

**Keywords:** Right aortic arch, tracheal stenosis, tuberculous sequelae, vascular bypass surgery, vascular tracheobronchial compression

## Abstract

A 74‐year‐old man, who had undergone thoracoplasty for tuberculous sequelae 54 years earlier, was referred to our hospital with a chief complaint of dyspnea. He had recently received mechanical ventilation due to pneumonia. However, although the pneumonia had improved, extubation was prevented by the presence of hypercapnic respiratory failure with tracheal stenosis due to compression of the right aortic arch and the left common carotid artery. Bypass surgery was performed, during which the left subclavian artery was placed over the left common carotid artery. Surgery resulted in expansion of the cross‐sectional tracheal stenosis area from 11.60 mm^2^ to 62.62 mm^2^, and the patient was successfully weaned off ventilatory support.

## Introduction

Benign tracheal stenosis is most commonly caused by congenital vascular anomalies. A much rarer cause is termed vascular tracheobronchial compression syndrome, which primarily occurs in adults 1. Knight et al. investigated 74 cases involving compression by the right aortic arch, and classified the patterns according to the arch branching 2. Here we describe a previously unreported pattern involving tracheal compression between the right aortic arch and the left common carotid artery, which occurred in a patient who had undergone thoracoplasty for tuberculous sequelae 54 years earlier. Vascular bypass surgery in this case of vascular tracheobronchial compression syndrome enabled successful ventilator weaning.

## Case Report

A 74‐year‐old male, who had undergone thoracoplasty for lung tuberculosis 54 years ago, presented at a hospital with a chief complaint of dyspnea. The patient was a nonsmoker and his activities of daily living were unhindered. While waiting to be examined, he began to feel ill and quickly lost consciousness. He received emergency mechanical ventilation and was diagnosed with pneumonia and treated with antibiotics. Six days later, his condition was improved, and ventilatory support weaning was attempted. However, after 8 h of weaning, the patient was re‐intubated due to CO_2_ narcosis. The patient was diagnosed with hypercapnic respiratory failure and was transferred to our hospital.

**Figure 1:**
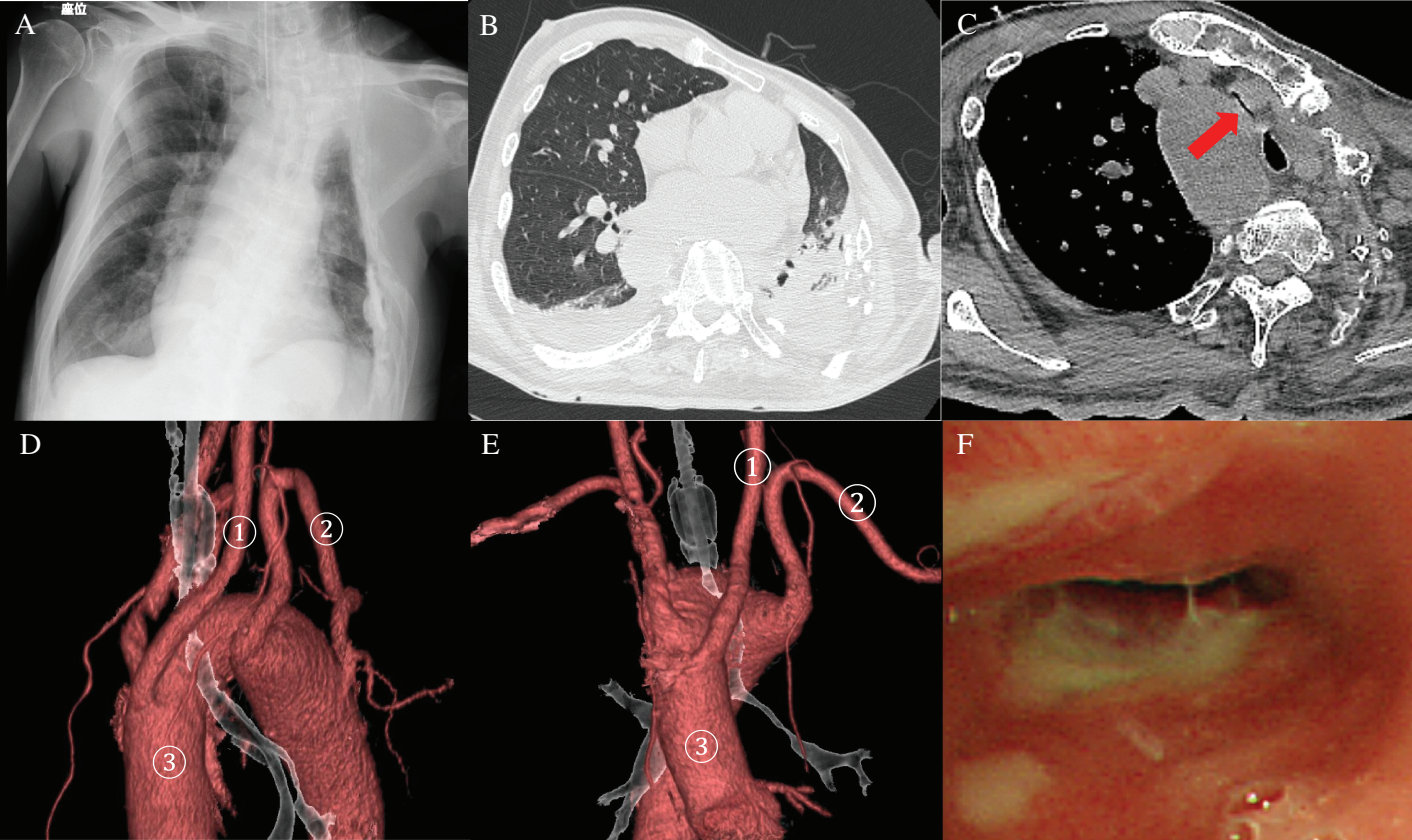
(A) Chest X‐ray showed infiltrative opacities in the lower fields of the left lung. (B) Chest CT (lung window) revealed an infiltrative shadow in the left lower lobe. (C) Chest CT (mediastinal window) showed tracheal compression between the right aortic arch and left common carotid artery with slit‐shaped stenosis (arrow). Using customizable CT image software (DICOM Viewer‐EV Insite R; PSP Corporation, Tokyo, Japan), we estimated a 96.5% stenosis rate with compression of the cross‐sectional area to 11.60 mm^2^ compared to 3 cm^2^ in the upper tracheal segment. (D, E) Chest CT with 3‐dimensional image reconstruction revealed that the trachea was compressed between the right aortic arch(③), and the left common carotid artery with an aberrant origin(①). ②: the left subclavian artery. (F) Bronchoscopic findings revealed redness and partial ulceration of the bronchial epithelium in the area of constriction.

Physical examination upon admission revealed consciousness of E4VTM6, a respiratory rate of 21/min, and audible bilateral coarse crackles in the lungs. Arterial blood oxygen saturation was 94% (CPAP mode: FiO2, 0.3; PS, 10 cm H_2_O; PEEP, 5 cm H_2_O). Under these ventilator conditions, arterial blood gas showed a pH of 7.473, PaO_2_ of 79.0 Torr, PaCO_2_ of 46.3 Torr, and HCO_3_− of 33.6 mmol/L. Chest X‐ray revealed infiltrative opacities in the lower field of the left lung (Fig. 1A). Chest computed tomography (CT) showed an infiltrative shadow in the left lower lobe, and severe tracheal compression between the right aortic arch and left common carotid artery (Fig. 1B, C). Using customizable CT image software, we estimated a 96.5% stenosis rate with compression on a cross‐sectional area of 11.60 mm^2^ compared to 3 cm^2^ in the upper segment of the trachea (Fig. 1C, D, E). Bronchoscopy revealed redness and ulceration of the endobronchial surface of the obstructing segment (Fig. 1F). The tracheal stenosis was an extraluminal type caused by compression by the major arteries.

Since stent placement would carry a risk of tracheal fistulation and massive bleeding, we decided to perform artery bypass surgery to release tracheal compression by placing the left subclavian artery over the left common carotid artery. Six days post‐operatively, the endoluminal ulceration improved and the obstruction gradually expanded. Seven days postoperatively, arterial blood gas had a pH of 7.473, PaO_2_ of 81.2 Torr, PaCO_2_ of 63.7 Torr, and HCO_3_− of 36.3 mmol/L (FiO2, 0.3; PS, 5 cm H_2_O; PEEP, 4 cm H_2_O).

**Figure 2:**
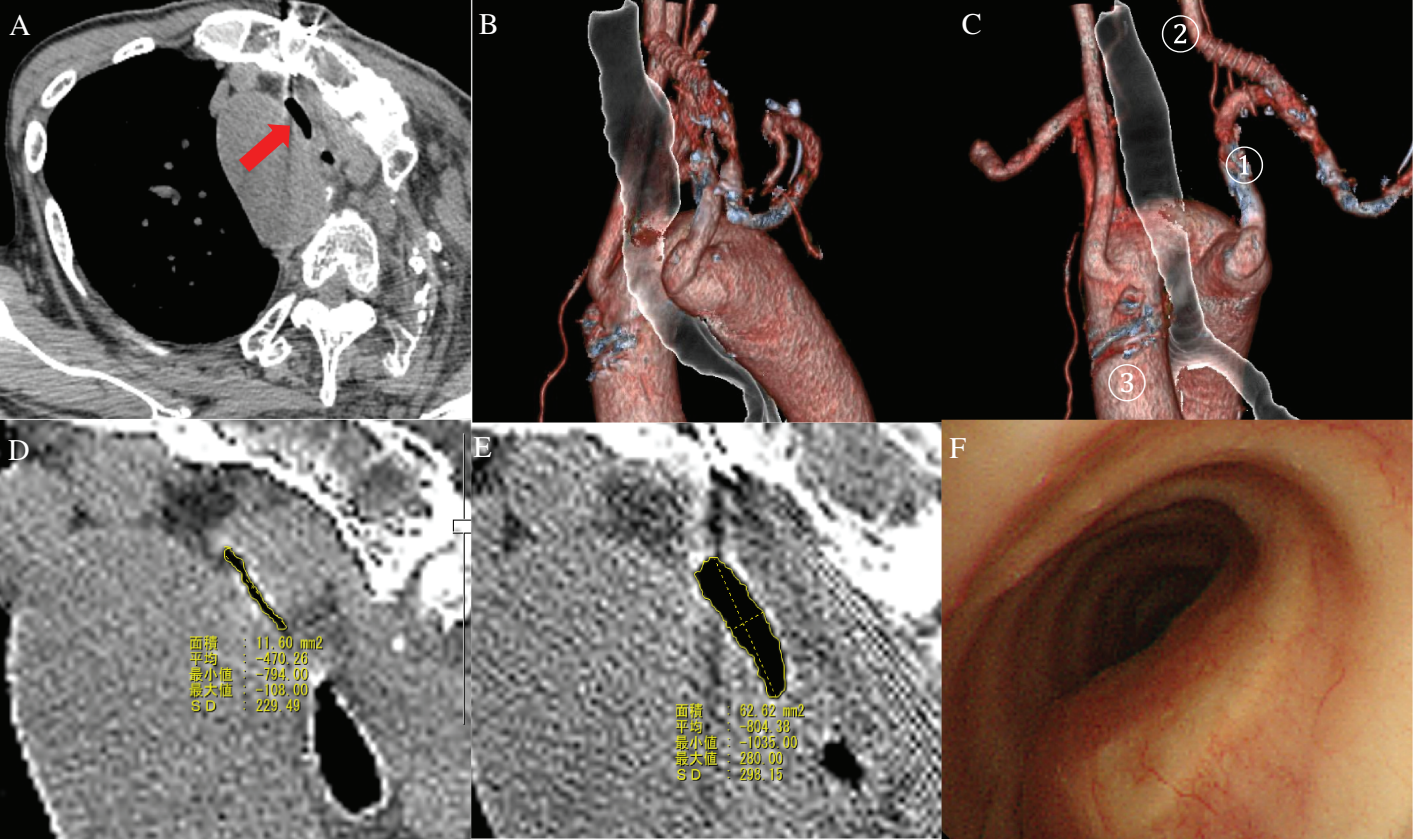
(A) Chest CT (mediastinal window) at 29 days after the bypass operation revealed expansion of the sectional area of the tracheal stenosis (arrow). (B, C) Chest CT with 3‐dimensional image reconstruction at 29 days after the bypass operation showed tracheal lumen dilation. (①: the left common carotid artery, ②: the left subclavian artery, ③: the right aortic arch.) (D) Chest CT (mediastinal window) revealed a pre‐operative sectional area of the tracheal stenosis of 11.60 mm^2^. The airway cross‐sectional area was measured using CT image customizable software (DICOM viewer‐EV insite R; PSP corporation, Tokyo, Japan). The most constricting area was manually indicated on the image. If the image of the most constricting area was indicated obliquely, the image was reconstructed to horizontally show the most obstructing area. The area was then calculated by tracing the most obstructing area in EV insite R. The percentage of the airway constriction was calculated by comparing the minimum cross‐sectional area with the cross‐ sectional area of nearby trachea (3 cm upper the constricted portion). A LungPoint® view is shown. The VBN system automatically indicated the distance to the peripheral end and proximal end of the target lesion. It also indicated the distance between virtual tip of the bronchoscopy and bronchial wall of the target in the view. From the view of this figure, the proximal end of the target is 7 mm, the peripheral end of the target is 15 mm, and the distance to the target bronchial mucosa is 4.3 mm. The puncture point is marked by white point in this view (i.e. center of the traced target lesion). (E) Chest CT (mediastinal window) revealed a post‐operative sectional area of the tracheal stenosis of 62.62 mm^2^. (F) Bronchoscopic findings at 34 days after bypass surgery showed improved healing of bronchial ulceration and maintenance of tracheal lumen dilation.

The patient was then weaned off ventilatory support. After 29 days, chest CT showed a trachea sectional area of 62.62 mm^2^, compared to 11.60 mm^2^ preoperatively (Fig. 2A, B, C, D, E). After 34 days, bronchoscopy revealed healing of bronchial ulceration, and maintained tracheal lumen dilation (Fig. 2F). The patient was discharged from our hospital at 46 days after bypass surgery.

## Discussion

Here we report a case of tracheal obstruction involving compression by the right aortic arch and left common carotid artery. The patient underwent bypass surgery in which the left subclavian artery was placed over the left common carotid artery.

Vascular tracheobronchial compression in adults can be congenital or acquired 3. Vascular anomalies of the right aortic arch are reportedly responsible for 55–78% of cases of tracheobronchial compression 3. Right aortic arch anomalies have been categorized into two types: with or without involvement of the retroesophageal aortic segment. Moreover, right aortic arch anomalies without retroesophageal aortic segment involvement are subdivided into three patterns based on the origin of the arch branches in the classification by Knight and Edwards 2.

The vascular anomaly in our present case was classified as a subtype of right aortic arch anomaly without retroesophageal aortic segment involvement and with involvement of an aberrant left subclavian artery. The thoracoplasty for the treatment of pulmonary tuberculosis led to development of prominent upper thoracic deformation, consequently, a mediastinal shift could gradually occur. The anterior parts of the trachea are usually compressed by the ascending aorta. However, in this case, the trachea was shifted to the left due to the previous thoracoplasty, such that it was compressed by the right aortic arch and left common carotid artery (Fig. 1A, C, D, E).

Attempts at ventilator weaning were thwarted by the tracheal compression and the increased secretion due to pneumonia.

Kanabuchi et al. suggest that bypass surgery alone is insufficient to resolve tracheal compressions, stating that tracheal stenosis treatment requires tracheal reinforcement, such as stent placement 3. However, the present case did not require stent placement, possibly because the patient had no bronchomalacia and exhibited sufficient tracheal dilation after surgery. In this case, stent placement may have resulted in massive hemorrhage due to fistula formation 4. Bypass surgery is probably the most appropriate treatment for patients with extraluminal tracheal stenosis due to vascular anomalies, especially in elderly patients who face greater risks with stent placement. To our knowledge, this is the first case report describing tracheal stenosis due to compression by the right aortic arch and left common carotid artery, and with treatment via artery bypass surgery.

### Disclosure Statement

Appropriate written informed consent was obtained for publication of this case report and accompanying images.
